# Supplementary material for: The Resistance of Maize to Ustilago maydis Infection Is Correlated with the Degree of Methyl Esterification of Pectin in the Cell Wall
Source: Int J Mol Sci. 2023 Sep 29;24(19):14737. doi: 10.3390/ijms241914737 (PMC10573042; doi:10.3390/ijms241914737)
Supplement: Supplementary file 1 [file ijms-24-14737-s001.zip › Supplementary Figures.pdf]

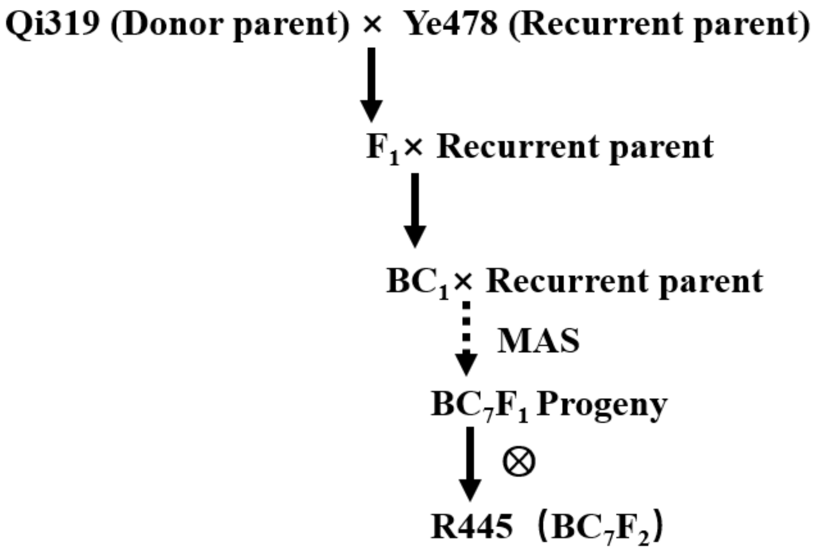

Figure S1. The diagrammatic representation of the genetic relationship between Ye478 and R445.

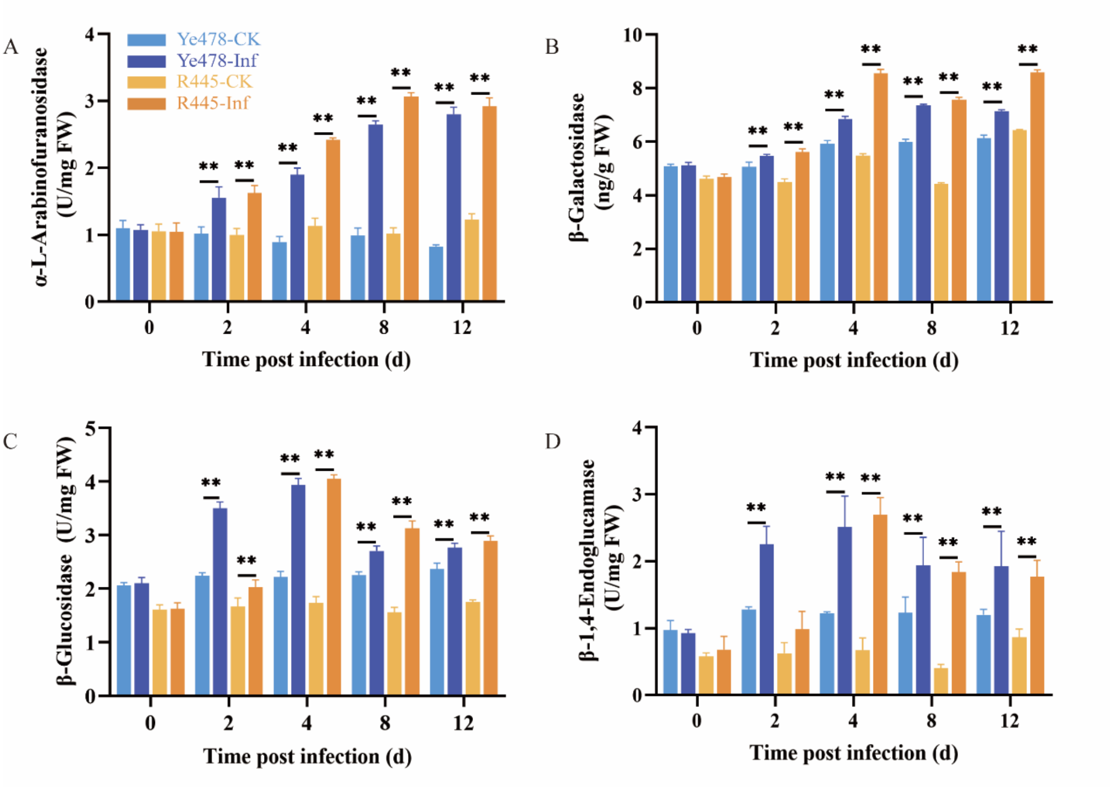

Figure S2. The changes in the contents of cell-wall-degrading enzymes (CWDEs) in the leaves infected by *U. maydis*. (A) α-L-arabinofuranosidase, (B) β-galactosidase, (C) β-glucosidase, and (D) β-1,4-glucanase contents were analyzed in maize leaves infected by *U. maydis*. Samples of Ye478 and R445 were collected at 0, 2, 4, 8, and 12days postinfection (dpi). Ye478-CK/R445-CK: leaves infected with ddH_2O; Ye478-Inf/ R445-inf: Ye478 leaves infected with *U. maydis*. Student's t test, * and ** represent significant difference at P ≤ 0.05 and ≤ 0.01, respectively.

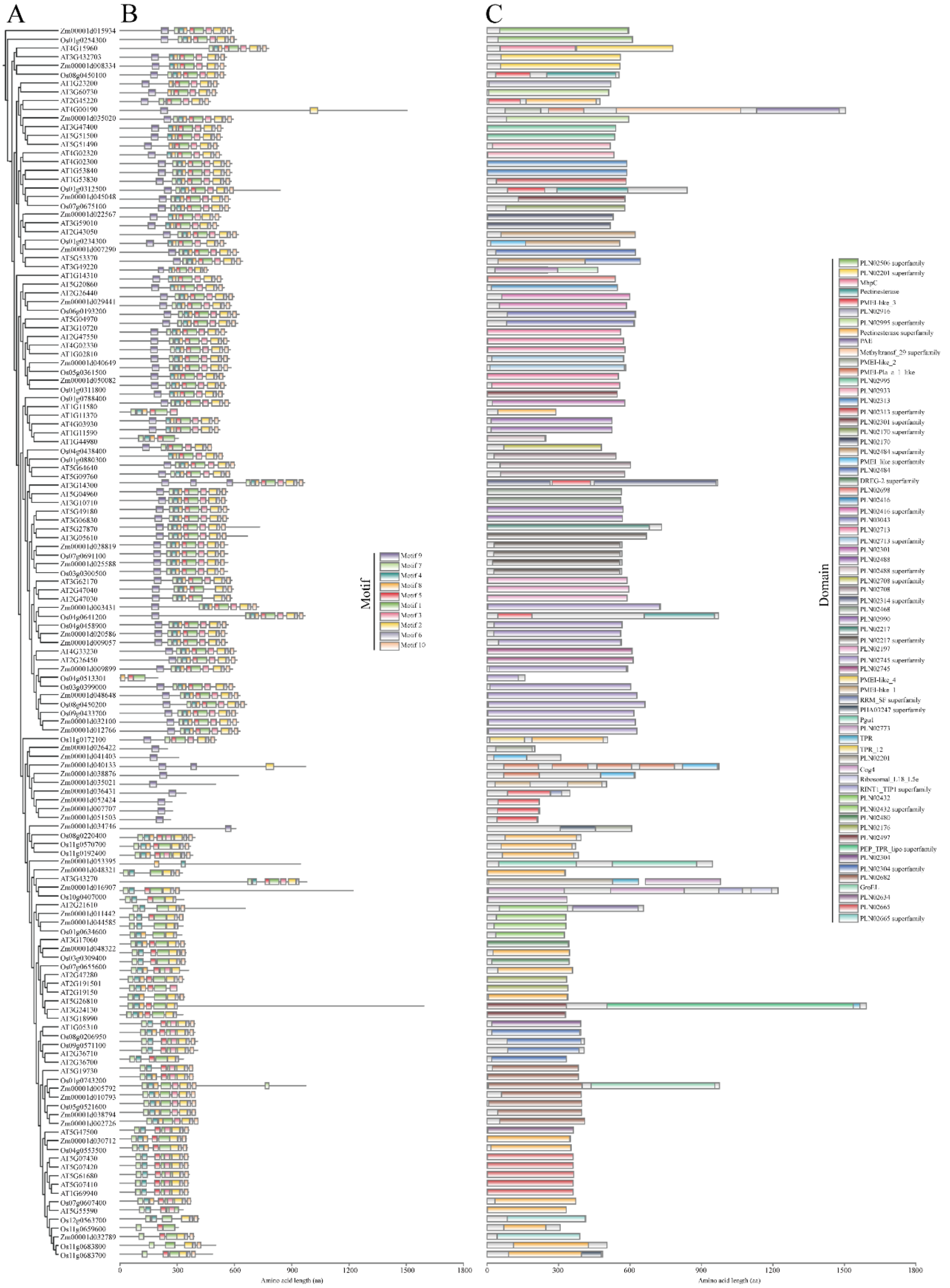

Figure S3. Phylogenetic relationships, conserved motifs, and domain structures of pectin methylesterase proteins in maize, rice, and *Arabidopsis*. (A) Phylogenetic tree of pectin methylesterase proteins in maize, rice, and *Arabidopsis*. (B) The composition and distribution of conserved motifs of pectin methylesterase proteins. (C) The composition and distribution of

domain structures of pectin methylesterase proteins.
