# Supplementary material for: The Resistance of Maize to Ustilago maydis Infection Is Correlated with the Degree of Methyl Esterification of Pectin in the Cell Wall
Source: Int J Mol Sci. 2023 Sep 29;24(19):14737. doi: 10.3390/ijms241914737 (PMC10573042; doi:10.3390/ijms241914737)
Supplement: Supplementary file 1 [file ijms-24-14737-s001.zip › Table S1.pdf]

Table S1. The genetic information of single segment substitution line (SSSL) R445

Primer	Primer sequence (5'-3')	Chromsomnal location (bins)	Genetic location (cM)	Physical location (bp)
umc1528	F: AGTTCAACTGCTTAAGATCCGGTG<br>R: GTCTGTCGTTGTGTGCCAGTG	3.07	544.4	198,039,220 198,039,356
umc1489	F: TTAATAGCTACCCGCAACCAAGAA<br>R: CTGAGCCACAGTACCTTGCTGTT	3.07	567.7	201,987,448 201,987,552
