# Supplementary material for: The Resistance of Maize to Ustilago maydis Infection Is Correlated with the Degree of Methyl Esterification of Pectin in the Cell Wall
Source: Int J Mol Sci. 2023 Sep 29;24(19):14737. doi: 10.3390/ijms241914737 (PMC10573042; doi:10.3390/ijms241914737)
Supplement: Supplementary file 1 [file ijms-24-14737-s001.zip › Table S2.pdf]

Table 2. The information of 15 maize inbred lines.

Number	Resistance level	Name of the inbred line	Heterotic groups
1.	susceptible	Dong6002	SPT
2.		HuangC	Reid
3.		KL4	Lan
4.		P007	PB
5.		391	Mixed
6.	partially susceptible	Xin444	SPT
7.		Dan598	LRC
8.		1028	Mixed
9.		Longxi69	LRC
10.		Cheng351	Mixed
11.	resistant	444	SPT
12.		K12	SPT
13.		Ji1037	Lan
14.		Dan6263	PB
15.		Sui601	Mixed
