# Supplementary material for: The Resistance of Maize to Ustilago maydis Infection Is Correlated with the Degree of Methyl Esterification of Pectin in the Cell Wall
Source: Int J Mol Sci. 2023 Sep 29;24(19):14737. doi: 10.3390/ijms241914737 (PMC10573042; doi:10.3390/ijms241914737)
Supplement: Supplementary file 1 [file ijms-24-14737-s001.zip › Table S3.pdf]

Table S3 Primer sequences and gene annotation for qRT-PCR.

Gene ID	Primer sequence (5′-3′)		Gene annotation	Gene origin
Zm00001d009899	F: TCAAACAAGCTCAGGGGCAA	R: GAGCGTAAGGCCTGAGAAGG	Pectin methylesterase17	Transcriptome data (Zou et al., 2022)
Zm00001d009057	F: GCTCTATGGTCAGTGCTGGA	R: CATAGGCCCCTCTTCCAGGT	Polygalacturonase49	Transcriptome data (Zou et al., 2022)
Zm00001d040649	F: CTGGACGACCTTCAACTCCG	R: GTAGAACGTCGAGAGGTCCG	Pectin methylesterase16	Transcriptome data (Zou et al., 2022)
Zm00001d051503	F: CCCCGATGACACCTATCCC	R: CATGTCATCAACATCGCGTCC	Pectinesterase inhibitor 12	Common data base (NCBI)
Zm00001d030712	F: GGCGGCGTTCTATGACTGTA	R: CCGAGGTAGAGTGCAAGTGG	Putative pectinesterase 11	Common data base (NCBI)
Zm00001d010793	F: ACATCACCTTCAAGCAGAACAC	R: GTAGCAGTCGCGGTAGTAGT	Pectin methylesterase 38	Common data base (NCBI)
Zm00001d028819	F: GCAATCGGGCAGGATACACT	R: TCATATCGACAACGCAGCCT	Putative pectinesterase	Common data base (NCBI)
GAPDH	F: CTTCGGCATTGTTGAGGGTTTG	R: TCCTTGGCTGAGGGTCCGTC	Zea mays *GAPDH* gene	-
ppi	F: AAAGAACACCGGACTTGG	R: ACATCGTCAAGGCTATCG	UMAG_03726	(Lin et al., 2021)

Note: Transcriptome data derived from Zou et al. (2022) and common data base using NCBI (https://www.ncbi.nlm.nih.gov/); "-" no data.
